# Resistance to Critical Important Antibacterials in *Staphylococcus pseudintermedius* Strains of Veterinary Origin

**DOI:** 10.3390/antibiotics11121758

**Published:** 2022-12-05

**Authors:** Alessandro Bellato, Patrizia Robino, Maria Cristina Stella, Laura Scarrone, Daniela Scalas, Patrizia Nebbia

**Affiliations:** Microbiology and Infectious Diseases Unit, Department of Veterinary Sciences, University of Turin, Largo Braccini 2, 10095 Grugliasco, Italy

**Keywords:** *Staphylococcus pseudintermedius*, antimicrobial resistance, critically important antimicrobials, antimicrobial susceptibility testing, cat, dog, Italy

## Abstract

Staphylococcal infections represent a challenge in companion animals and hospitalized patients. This study aimed to assess the resistance of *Staphylococcus pseudintermedius* isolates, against a broad panel of antibacterials, including exclusive to human medicine. A total of 40 *S. pseudintermedius* were collected from clinical specimens of dogs (*n* = 31) and cats (*n* = 5). All strains were tested for 20 antibacterials, namely 14 Critical Important and eight Highly Important Antibacterials (CIA and HIA, respectively), indicative for 18 antimicrobial classes. All strains were susceptible to seven antibiotics (daptomycin, fosfomycin, fusidic acid, linezolid, quinupristin-dalfopristin, teicoplanin/vancomycin, tigecycline). The highest resistance was against penicillin (97.5% Confidence Interval [CI]: 83.8–100.0), whereas the lowest against telavancin (2.5%, CI: 0.0–16.2). Resistance versus Highest Priority CIA was observed, namely against macrolides (70.0, CI: 52.1–84.3), quinolones (62.5, CI: 44.5–78.3), 5th generation cephalosporins (7.5, CI: 1.3–21.6), and glycopeptides (2.5%, CI: 0.0–14.2). Among High Priority CIA, strains were resistant only to aminoglycosides (65.0, CI: 47.0–80.4) and ansamycins (12.5, CI: 3.8–28.1). We observed the highest resistance against veterinary medicine antibacterials, but there was also resistance against antibacterials exclusive to human medicine, namely ceftaroline (7.5, CI: 1.0–23.8) and telavancin. *S. pseudintermedius* zoonotic potential and its rate of acquisition of new resistance should encourage surveillance on a broad spectrum of antibacterials.

## 1. Introduction

*Staphylococcus pseudintermedius* was first discovered in 2005 and rapidly gained plenty of fame due to its pathogenic potential [1]. To date, there are more than 700 articles on PubMed [2] on this topic. In dogs, *S. pseudintermedius* is present as a normal inhabitant of the skin [3] but also as an opportunistic pathogen that causes skin, ear, and surgical-site infections. In cats, it has a lower prevalence [4], and some authors suggested that it could be due to the antagonism by *Staphylococcus felis* [5]. It is preferentially transmitted via direct animal–animal contact, but it has been reported to infect humans through dog–human transmission [6,7,8]. The first human case was reported in 2006; growing evidence has been pointing to this bacterium as a human pathogen of concern especially in elderly, diabetic and immunocompromised patients or in people that work in close proximity to animals [9]. Similar to other coagulase-positive staphylococci, *S. pseudintermedius* typically expresses various virulence factors (e.g., coagulase, cytotoxin, enterotoxin, hemolysin, leukocidins, proteases, thermonuclease, etc.) [10] and can acquire resistance to a variety of antibiotics via the acquisition of resistance genes (e.g., *blaZ* and *mecA* for β-lactams, *tetK* and *tetM* for tetracyclines, *erm* for erythromycin, etc.) [11]. Since its discovery, it has been of increasing concern due to its high genetic heterogeneity, which makes it hard to type [12]. Companion animals sharing the same environment with their owners can transmit not only pathogens but resistance genes as well [13,14]. In recent years, the EU legislation curtailed the number of antibacterials available for veterinary prescription to lower the risk of antimicrobial resistance (AMR) derived from treatment in veterinary medicine [15]. With a similar aim, the World Health Organization (WHO) developed the criteria to rank antibacterials depending on their importance in human medicine [16,17]. The list of antimicrobials important for human medicine—first presented in Canberra in 2005—was revised multiple times until 2018. The purpose of this document was to increase the awareness of public and animal health authorities, physicians, and veterinarians about the prudent use of antimicrobials, especially the critically important. To draft it, two different criteria were implemented: (C1) “The antimicrobial class is the sole, or one of limited available therapies, to treat serious bacterial infections in people”, and (C2) “The antimicrobial class is used to treat infections in people caused by either: (1) bacteria that may be transmitted to humans from non-human sources, or (2) bacteria that may acquire resistance genes from non-human sources” [17]. Following these criteria, antimicrobials were defined as critically important (CIA) when they meet both C1 and C2, highly important (HIA) if they meet either C1 or C2, or important (IA) when neither C1 nor C2 is met. Three further prioritization factors were applied to subdivide CIA having high (HCIA) or the highest priority (HPCIA), depending on the amount of evidence that shows frequent transmission of resistant bacteria or genetic elements.

In 2017, WHO developed the Access, Watch, Reserve (AWaRe) classification of antibacterials to emphasize the importance of their prudent use [18]. More than 250 antimicrobials have been classified into three groups, Access, Watch and Reserve, depending on their relevance for antimicrobial resistance. The Access group includes antibacterials with low resistance potential and are active on a wide range of susceptible pathogens; the Watch group are antibacterials with a higher resistance potential, most of them being CIA; the Reserve group includes the antibacterials that should be used only as last resort options to treat confirmed or suspected infections due to multi-drug-resistant organisms [18].

Since 2014, the European Medicines Agency (EMA) expert group has been updating the categorization of antibacterials for veterinary medicine, acknowledging the WHO’s CIA list. This categorization was intended to promote the responsible use of antibacterials to protect public and animal health [15]. It subdivides antimicrobials into four categories: cat. A “Avoid” includes those not authorized in veterinary medicine and exclusive to human medicine; cat. B “Restrict” includes all HPCIA not in cat. A except for macrolides; cat. C “Caution” are antibacterials with an alternative in human medicine but still at risk of developing resistance to a substance in cat. A, or without a less hazardous alternative in veterinary medicine; cat. D “Prudence” includes antibacterials with available alternatives in veterinary medicine that do not select for resistance to cat. A substances. It is noteworthy that cat. A comprehends all newly discovered substances, which may be used only for individual treatment in companion animals and under exceptional circumstances.

Reducing the number of antibiotics available for veterinary prescription determined the concomitant reduction in the number of antimicrobial susceptibility tests performed for clinical purposes in many veterinary laboratories. However, since *S. pseudintermedius* is of concern also for public health, it is of utmost importance to monitor the progress of its resistance pattern toward a broad panel of antibacterials that includes cat. A substances as well. However, one of the main problems of antimicrobial susceptibility testing (AST) in veterinary medicine is the absence of reliable clinical breakpoints for many pathogen-species-administration route combinations. The paucity of veterinary-specific data forces laboratories to refer alternatively to standards provided for human medicine by the Clinical Laboratory Standard Institute (CLSI) or the European Committee for AST (EUCAST).

This study aimed to assess the resistance of zoo-pathogenic *S. pseudintermedius* strains against a broad spectrum of antimicrobials of many different classes, including some exclusive to human medicine. 

## 2. Results

All the strains were resistant to at least one antibiotic. They were all susceptible to seven antibacterials, namely tigecycline, fosfomycin, fusidic acid, linezolid, teicoplanin/vancomycin, daptomycin, and streptogramins. The highest resistance was observed against penicillin since all but one isolates were resistant (97.5%, CI: 88.7–99.9). The lowest was reported for glycopeptides, with only one strain resistant to telavancin (2.5%, CI: 0.1–11.3). Resistance higher than 50% was recorded for six classes of antibacterials, namely against macrolides and lincosamides (70.0%, CI: 56.0–81.7%), aminoglycosides (65.0%, CI: 50.8–77.4%), fluoroquinolones (62.5%, CI: 48.3–75.3%), sulfamethoxazole-trimethoprim (60.0%, CI: 45.8–73.1%), and tetracyclines (57.5%, CI: 45.8–73.1%). Lower resistance was observed towards chloramphenicol (27.5%, CI: 16.3–41.4%) and rifampicin (12.5%, CI: 5.1–24.5%).

Among the 39 strains resistant to penicillin, 24 (61.5%) were resistant to oxacillin, thus being classified as methicillin-resistant *S. pseudintermedius* (MRSP). Furthermore, out of 24 MRSP, 3 (12.5%) were resistant (R) to 5th generation cephalosporins and another 3 were susceptible to increased dosage (I) only (Table 1).

Resistance versus HPCIA was observed, namely against macrolides, quinolones, 5th-generation cephalosporins, and glycopeptides. Regarding HCIA, strains were resistant only to aminoglycosides and ansamycins. Up to four strains were resistant against antibacterials in the Reserve category, namely three to ceftaroline and one to telavancin. A similar situation is reported for cat. A antibacterials, where four strains were resistant to rifampicin, two to ceftaroline, one to telavancin, and one to rifampicin and ceftaroline (Table 2). 

**Figure 1:**
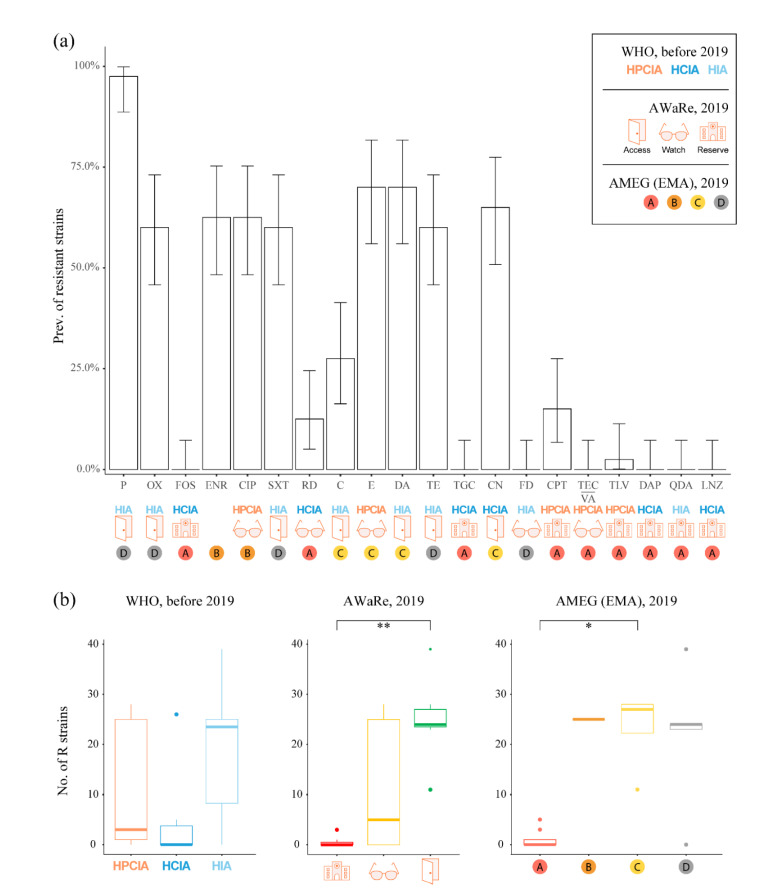
Bar plot of the prevalence of resistant strains (**a**). The height of the bars represents the point estimate prevalence of resistantstrains (R) to each antibacterial, with error bars indicating the 90% confidence intervals. Under the antibacterial name, World Health Organization (WHO and AWaRe) and European Medicines Agency (AMEG) classifications are reported. Box plots of the frequency of R strains (**b**). Data are aggregated by WHO, AWaRe and AMEG categories (*: *p* < 0.05; **: *p* < 0.01). Category abbreviations: HPCIA: Highest Priority, Critically Important Antimicrobials; HCIA: High priority, Critically Important Antimicrobials; HIA: Highly Important Antimicrobials. Antibacterial abbreviations: P: penicillin G; OX: oxacillin; FOS: fosfomycin; ENR: enrofloxacin; CIP: ciprofloxacin; SXT: trimethoprim-sulfamethoxazole; RD: rifampicin; C: chloramphenicol; E: erythromycin; DA: clindamycin; TE: tetracycline; TGC: tigecycline; CN: gentamycin; FD: fusidic acid; CPT: ceftaroline; TEC/VA: teicoplanin/vancomycin; TLV: telavancin; DAP: daptomycin; QDA: quinupristin-dalfopristin; LNZ: linezolid. Note: the colours are those used by WHO and EMA to identify the categories of antibacterials.

There was no difference among WHO categories since resistance to CIA quite matched HIA (Figure 1a). Regarding the AWaRe classification, the highest prevalence of resistant strains was observed against Access antibacterials, followed by Watch and Reserve (Figure 1b). The prevalence of strains resistant to Access antibacterials was significantly higher than those of the Reserve (*p* = 0.005). Regarding AMEG classification, the prevalence of strains resistant to cat. A antibacterials was significantly lower, compared to cat. C (*p* = 0.027). However, up to 12.5% prevalence was observed for rifampicin and 7.5% for ceftaroline, also with three strains susceptible to increased dosage (I). Four out of five cat. D antibacterials had a point-estimated resistance of over 50% with the only exception of fusidic acid.

Nineteen different antimicrobial-resistance (AMR) profiles (from A to S) were identified, which are resistant to a median of 8.5 antibacterials (mean ± standard deviation = 6.9 ± 3.5). A total of 2 of them were resistant to 11 antibacterials, 3 to 10, 5 to 9 and 1 to 8, whereas 8 were resistant to no more than 6 antibacterials each. Among them, only profile K was resistant to telavancin, along with penicillin and gentamycin. The highest frequency was recorded for profile B (freq = 7), which was susceptible to all antibacterials but penicillin, followed by profile E (freq. = 6, resistance to 9 antibacterials), C (freq. = 3, resistance to 8 antibacterials) and F (freq. = 3, resistance to 10 antibacterials). 

Eleven profiles (57.9%) were attributable to MRSP. Except for Q, the other profiles of methicillin-susceptible *S. pseudintermedius* (MSSP) were resistant to no more than six antibacterials. Accordingly, MRSP strains were significantly (*p* < 0.0001) resistant to more antibacterials (median = 9, mean ± standard deviation = 8.9 ± 2.3) than MSSP strains (median = 2, mean ± s.d. = 3.3 ± 2.9). MRSP strains were significantly more resistant to aminoglycosides (*p* < 0.0001), macrolides and lincosamides (*p* < 0.001), quinolones (*p* < 0.0001), sulfamethoxazole-trimethoprim (*p* < 0.001), and tetracyclines (*p* = 0.0245). The proportion of strains resistant to chloramphenicol was not significantly higher in MRSP than in MSSP (*p* = 0.4732), and it was identical for rifampicin.

Resistance to ceftaroline was observed in profiles D, L, and M, which were resistant to 10, 9, and 11 antibacterials, respectively. (Table 3). 

Only 3 out of 19 AMR profiles (15.8%) were not multi-drug resistant (B, I, and P). Overall, the prevalence of MDR strains was 75.0% (CI: 61.3–85.8%). A total of 60.0% of the strains (CI: 45.8–73.1%) were multi-resistant to CIA (i.e., resistant to 3 or more classes of CIA), whereas only 20.0% (CI: 10.4–33.2%) of strains were multi-resistant to antibacterials in Watch and/or Reserve categories. The prevalence of strains resistant to three or more classes of cat. A, B or C combined was 70.0% (CI: 56.0–81.7%). No extensively drug-resistant (XDR) nor pan-drug resistant (PDR) strains were observed. The overall multiple antimicrobial resistance (MAR) index was 0.34, but it ranged from 0.18 in MSSP to 0.49 in MRSP strains.

## 3. Discussion

This study assessed the resistance of forty zoo-pathogenic *S. pseudintermedius* strains against a broad spectrum of antimicrobials of many different classes, including some exclusive to human medicine. The explorative aim of the study was more of epidemiologic than of clinical relevance. In fact, the applicability of the results to veterinary clinics is limited by the lack of several clinical breakpoints for *S. pseudintermedius*. However, for epidemiological purposes, it is important to monitor the resistance to human-specific antibacterials, in particular for bacterial species with zoonotic potential, such as S. pseudintermedius.

During a one-year period, all cases matching the inclusion criteria that occurred at the veterinary teaching hospital of the University of Torino were enrolled. From 36 animals, 40 *S. pseudintermedius* strains were isolated. The 36 cases came from different locations of the province of Torino in the north-west of Italy, thus representing the geographic area where the study was conducted. The sample size was satisfactory for a preliminary assessment in terms of prevalence of resistant strains, even if having collected cases over a longer period would have helped the generalizability of the results. The proportion of feline patients confirmed the low prevalence observed in cats [19] and the higher occurrence of *S. pseudintermedius* infection in dogs [20].

All strains were susceptible to seven antibacterials, although some were resistant to other antibacterials of the same classes. Noticeably, all strains were susceptible to fusidic acid, conversely more than what was reported elsewhere [10]. The prevalence of resistant strains is similar to a previous report in Italy [11] but higher than reported in other geographical areas [4,10,19,20,21]. Most strains were resistant to penicillin and up to 60% were resistant to oxacillin, a percentage higher than recently reported in dogs [10,20,21] and cats [19,20]. In staphylococci, methicillin-resistance is noticeable not only because it involves the resistance to many beta-lactams and cephalosporins but also because it is related to increased resistance towards other antibacterials [21]. Our results confirm these findings, since MRSP strains were more resistant to most of the tested antibacterials, apart from rifampicin and chloramphenicol. Moreover, on average, they were resistant to more antibacterials compared to MSSP. In addition, this was supported by AMR profiles and their frequency.

One third of the MRSP were resistant to ceftaroline as well, based on human-specific clinical breakpoints. This is of particular concern because 5th generation cephalosporins, especially ceftaroline, are currently regarded as the antibacterials of choice against difficult-to-treat multidrug-resistant Gram-positive organisms, such as methicillin-resistant staphylococci [22].

The strains resistant to aminoglycosides, macrolides and lincosamides, quinolones, sulfamethoxazole-trimethoprim, and tetracyclines were over 50%, again, percentages higher than recently reported in recent studies [19,20,21] but in line with others [10,11]. Accordingly, the 60%prevalence of multi-drug resistant strains was higher as well. We observed resistance to no more than 11 antibacterials per strain, thus no extensively drug resistant nor pan-drug resistant strains were detected. An overall MAR index of 0.34 indicated a moderate degree of multi-drug resistance. This was even higher in MRSP, which were resistant to nine antibacterials, on average. Analyzing the AMR profiles, it was possible to identify the most recurrent pattern of resistance. For example, the resistance to chloramphenicol, although having a low prevalence, was recorded only in MDR strains, in association with beta-lactams and macrolides resistance. As expected, resistance to ceftaroline was compounded by the fact that it is found in multi-resistant strains.

The AMR profile analysis, as well testing for a broad spectrum of antibacterials, allowed us to observe that the resistance was higher to antibacterials available in veterinary medicine, suggesting that the major determinant of AMR is the selective pressure exerted by the treatment [23]. Indeed, this finding does not rule out the hypothesis that sharing the same environment with humans leads to the selection of strains resistant to human-specific antibacterials. In fact, antimicrobial resistance in companion animals is known to be acquired not only through treatment but also via transmission back and forth with their owners, which occurs on a regular basis when sharing the same environment [13,14,24]. Moreover, the same antimicrobial classes are used in human medicine, too.

It was of particular interest to examine the results by antimicrobial categorization. The focus was on the EMA categories for veterinary antibacterials and the WHO categories for human antibacterials. Such categorizations have been useful in differentiating substances according to their relevance and in suggesting a reasonable cascade for prescribing [15]. In addition, AMEG classification contemplates the tendency of certain antibacterials to select strains resistant to other categories as a criterion of choice for the treatment. However, the AWaRe categorization appeared to best represent the observed resistance. In fact, the highest resistance was observed for *Access* substances, which are the most widespread and least relevant, while the lowest resistance was against the *Reserve* substances, which should be avoided except as a last resort.

Broadening the panel of tested substances has a rebound on the prevalence of MDR strains, in fact it increases the chance of finding them. Therefore, we additionally evaluated the prevalence of strains multi-resistant to “critical” antibacterials, which depended on the categorization. It ranged from 20% for *Watch* + *Reserve* categories up to 70% versus cat. A, B or C combined. Additionally, it is notable that there were very different levels of resistance versus cat. D antibacterials. In fact, all but one strains were resistant to benzylpenicillin, while all strains were susceptible to fusidic acid, which has recently made a comeback for the treatment of skin infections [25]. Eventually, even if the resistance to cat. A antibacterials was low, more than half the strains were at the same time resistant to most antibacterials in cat. D, C, and B.

## 4. Materials and Methods

### 4.1. Sampling

We hypothesized that *S. pseudintermedius* isolates from clinical specimens of dogs and cats harbored resistance to antibiotics that are exclusive to human medicine. To confirm this hypothesis, cases admitted to the Veterinary Teaching Hospital (VTH) of the Department of Veterinary Science of the University of Turin (Italy) from July 2019 to May 2020 were collected. Eligibility criteria for case enrolment were the admission to the VTH for bacteriological infection and the isolation of *S. pseudintermedius* alone or as the primary pathogen. After the isolation of *S. pseudintermedius*, the previous clinical history of the subjects was checked to exclude all patients that received antibacterial treatment in the previous 90 days.

Forty *S. pseudintermedius* strains were isolated from 36 animals of which 31 were dogs (86.1%) and 5 were cats (13.8%). In descending order of frequency, the reasons for admission were orthopedic surgeries, skin infections or wounds, ear infections or surgeries, urinary tract infections, and reproductive system infections (Table 4).

Overall, seven strains with diverse resistance profiles came from three different dogs. In July 2019, a dog was diagnosed with chronic pyoderma and a *S. pseudintermedius* strain was isolated: based on AST results, a fusidic acid ointment was administered locally for a month. At a second sampling, in March 2020, another strain of *S. pseudintermedius*, still susceptible to fusidic acid but with a different resistance profile, was isolated.

Two *S. pseudintermedius* strains were derived from a crossbred dog with external otitis. The first was isolated in November 2019 during an ear canal ablation surgery. It was treated with amoxicillin-clavulanate. Four months later, in April 2020, the second was obtained from a surgical revision due to the presence of an abscess. The two isolates were considered different from each other based on the AST.

On the 17 September 2019, a crossbred dog was admitted to the VTH for right femur fracture. During the orthopedic surgery for a hip replacement, the first strain of *S. pseudintermedius* was isolated. In January 2020, the dog was hospitalized for surgical site infection, and two different strains of S. pseudintermedius were isolated from a screw of the surgical implant and a swab. The strains showed different resistance profiles.

### 4.2. S. pseudintermedius Identification

The sampling procedure depended on the source of infection and was performed by the clinician during the examination. Samples were stored at 4 ± 2 °C and inoculated within 24 h from collection. *Staphylococcus* spp. strains were isolated by culture on Columbia colistin and nalicanic acid (CNA) blood agar (Oxoid, Basingstoke, UK) and identified to the species level by MALDI-TOF mass spectrometry. The samples were prepared for mass spectrometry following the guidelines of the manufacturer for extended Direct Transfer (eDT) procedure, e.g., transferring on single colony direct on steel plate using a toothpick, applying 1 μL of formic acid and allowing it to dry, then covering with α-cyano-4-hydroxycinnamic acid (HCCA) matrix and allowing it to dry completely. Due to the known difficulties in *Staphylococcus intermedius* group (SIG) species discrimination, MALDI scores above 1.60 were considered satisfactory if the first three or more species suggested by the algorithm were *S. pseudintermedius*. Eventually, the identification of all strains was confirmed by PCR for the *S. pseudintermedius* species-specific *nuc* gene [26].

### 4.3. Antimicrobial Susceptibility Testing

For this study, antibacterials were selected to represent as many classes as possible, starting from the list proposed by Magiorakos et al. for evaluating multi-drug resistance [27]. Among different antibacterials of the same class, the choice was made based on the availability of clinical breakpoints. Eventually, 20 antibacterials were tested (Table 5), namely 11 CIA (ceftaroline, ciprofloxacin, daptomycin, erythromycin, fosfomycin, gentamycin, linezolid, rifampicin, teicoplanin/vancomycin, telavancin, tigecycline) and 8 HIA (chloramphenicol, clindamycin, fusidic acid, oxacillin, penicillin G, quinuprstin-dalfopristin, tetracycline, trimethoprim-sulfamethoxazole), indicative of 18 different antimicrobial classes; 6 of them (ceftaroline, daptomycin, linezolid, quinupristin-dalfopristin, teicoplanina/vancomycin, telavancin) were tested by measuring minimum inhibitory concentration (MIC) with MIC-test strips (Liofilchem Inc., Waltham MA, USA) and the others (chloramphenicol, ciprofloxacin, clindamycin, enrofloxacin, erythromycin, fosfomycin, fusidic acid, gentamycin, oxacillin, penicillin, rifampicin, sulfamethoxazole-trimethoprim, tetracycline, tigecycline) by the agar disk diffusion (ADD) method. To interpret the MIC and inhibition halos diameters, clinical breakpoints of the European Committee of Antimicrobial Susceptibility Testing (EUCAST, 2019) [28] were used except for enrofloxacin for which we referred to the Clinical Laboratory Standard Institute for veterinary medicine (CLSI Vet, 2018) [29] and for fosfomycin, which was evaluated based on the breakpoints provided by the Comité de l’Antibiogramme de la Société Française de Microbiologie (CASFM, 2013) [30]. Following t’e manufacturer's instructions, MIC values, which fell between two-fold dilutions were rounded up to the next standard upper value. Some of the antibacterials are considered markers of resistance for other classes, such as oxacillin for cephalosporins from 1st to 4th generation and carbapenems, thus increasing the number of classes evaluated.

### 4.4. Statistical Analysis

Based on the results of AST, a multi-drug resistant (MDR) strain was defined as being resistant to at least one antibacterial in more than three antibacterial classes. Extensively drug-resistant (XDR) was defined as nonsusceptibility to at least one agent in all but two or fewer antibacterial classes. Strains resistant to at least one antimicrobial in all antimicrobial classes were defined as pan-drug resistant (PDR) [27]. Based on the most recent definition of susceptibility categories (S, I, and R) provided by EUCAST [31], S and I were both considered susceptible and lumped together to estimate the prevalence of resistant strains. The prevalence proportion was calculated as the number of isolates not susceptible to the antibacterial over the total number of isolates. After the evaluation of Wald’s, Wilson’s, and Clopper-Pearson’s confidence interval [32], the latter was chosen for being the most conservative.

The comparison between MRSP and MSSP strains was performed using Fisher’s test for contingency tables and Wilcoxon rank-sum test. The prevalence of resistant strains was compared among categories for each antibacterial categorization (WHO, AMEG, and AWaRe), using Fisher’s exact test for contingency tables and the Kruskal–Wallis test. For comparisons between two categories, we used a pairwise Wilcoxon rank-sum test with Bonferroni correction for multiple tests.

To evaluate the number of antibacterials to which strains were resistant, the Multiple Antimicrobial Resistance (MAR) index was calculated, as described by Krumperman [33]. To avoid the risk of MAR underestimation, only one antibacterial of the same class was included.

The selection process of some estimates to be presented in the abstract adds additional uncertainty, which confidence intervals do not account for. Therefore, we corrected the intervals accordingly [34].

## 5. Conclusions

Our findings confirm that the resistance of *S. pseudintermedius* strains was higher towards antibacterials commonly used in veterinary medicine than to human-exclusive substances. Nonetheless, some resistance to these last is present. Since *S. pseudintermedius* is a major cause of disease in companion animals and is increasingly reported in human patients as well, we would encourage the monitoring of its evolution, and the introduction of new categories of multi-drug resistant strains devoted to critically important antibacterials.

Based on the results of this study, there is no evidence that it is necessary to broaden the spectrum of antibacterials to be tested routinely. Nonetheless, it is essential to carefully consider many factors when choosing the appropriate treatment, not only the susceptibility of the strain. To this end, the classifications of antibacterials provide proper guidance, although the emergence of multi-resistant strains is making some of them obsolete. Regardless of a treatment being administered to an animal or a human being, it is necessary to reiterate the extreme importance of prudent antibiotic use since we all operate in a one-health context.

## Figures and Tables

**Table 1:** Results of antimicrobial susceptibility testing. Absolute frequency of sensitive (S), sensitive to increased dosage (I) and resistant (R) strains are reported along with the prevalence of R strains and 90% confidence intervals (CI) for each antibacterial tested.

Class/Group	Antibacterial	S (freq.)	I (freq.)	R (freq.)	R Prev. [CI] (%)
β-lactamase-sensitive penicillins	P	1	0	39	97.5 [88.7–99.9]
β-lactamase-resistant penicillins	OX	16	0	24	60.0 [45.8–73.1]
phosphonic acids	FOS	40	0	0	0.0 [0.0–7.2]
fluoroquinolones	ENR	15	0	25	62.5 [48.3–75.3]
	CIP	15	0	25	62.5 [48.3–75.3]
folate-pathway inhibitors	SXT	16	0	24	60.0 [45.8–73.1]
ansamycins	RD	35	0	5	12.5 [5.1–24.5]
amphenicols	C	29	0	11	27.5 [16.3–41.4]
macrolides	E	12	0	28	70.0 [56.0–81.7]
lincosamides	DA	12	0	28	70.0 [56.0–81.7]
tetracyclines	TE	16	1	23	57.5 [45.8–73.1]
glycylcyclines	TGC	40	0	0	0.0 [0.0–7.2]
aminoglycosides	CN	14	0	26	65.0 [50.8–77.4]
steroid antibacterials	FD	40	0	0	0.0 [0.0–7.2]
anti-MRS cephalosporins	CPT	34	3	3	7.5 [6.7–27.5]
glycopeptides	TEC/VA	40	0	0	0.0 [0.0–7.2]
	TLV	39	0	1	2.5 [0.1–11.3]
lipopeptides	DAP	40	0	0	0.0 [0.0–7.2]
streptogramins	QDA	40	0	0	0.0 [0.0–7.2]
oxazolidinones	LNZ	40	0	0	0.0 [0.0–7.2]

Abbreviations: P: penicillin G; OX: oxacillin; FOS: fosfomycin; ENR: enrofloxacin; CIP: ciprofloxacin; SXT: trimethoprim-sulfamethoxazole; RD: rifampicin; C: chloramphenicol; E: erythromycin; DA: clindamycin; TE: tetracycline; TGC: tigecycline; CN: gentamycin; FD: fusidic acid; CPT: ceftaroline; TEC/VA: teicoplanin/vancomycin; TLV: telavancin; DAP: daptomycin; QDA: quinupristin-dalfopristin; LNZ: linezolid.

**Table 2:** Results of antimicrobial susceptibility tests aggregated by World Health Organization (WHO and AWaRe) and European Medicines Agency (AMEG) classifications of antibacterials. The absolute frequency of sensitive (S), sensitive to increased dosage (I), and resicannt (R) strains is reported along with the prevalence of R strains with 90% confidence intervals (CI).

Classification	Category	S	I	R	Prev. [CI] (%)	Antibacterials
WHO *	HPCIA	140	3	57	28.5 [23.3–34.2]	CIP, E, CPT, TEC/VA, TLV
	HCIA	209	0	31	12.9 [9.5–17]	FOS, RD, TGC, CN, DAP, LNZ
	HIA	170	1	149	46.6 [41.9–51.3]	P, OX, SXT, C, DA, TE, FD, QDA
AwaRe *	Access	104	1	175	62.5 [57.5–67.3]	P, OX, SXT, C, DA, TE, CN
	Watch	142	0	58	29.0 [23.7–34.7]	CIP, RD, E, FD, TEC/VA
	Reserve	273	3	4	1.4 [0.5–3.2]	FOS, TGC, CPT, TLV, DAP, QDA, LNZ
AMEG	A	348	3	9	2.5 [1.3–4.3]	FOS, RD, TGC, CPT, TEC/VA, TLV, DAP, QDA, LNZ
	B	30	0	50	62.5 [52.7–71.6]	ENR, CIP
	C	67	0	93	58.1 [51.3–64.7]	C, E, DA, CN
	D	89	1	110	55 [48.9–61.0]	P, OX, SXT, TE, FD

Category abbreviations: HPCIA: Highest Priority, Critically Important Antimicrobials; HCIA: High priority, Critically Important Antimicrobials; HIA: Highly Important Antimicrobials. Antibacterial abbreviations: P: penicillin G; OX: oxacillin; FOS: fosfomycin; ENR: enrofloxacin; CIP: ciprofloxacin; SXT: trimethoprim-sulfamethoxazole; RD: rifampicin; C: chloramphenicol; E: erythromycin; DA: clindamycin; TE: tetracycline; TGC: tigecycline; CN: gentamycin; FD: fusidic acid; CPT: ceftaroline; TEC/VA: teicoplanin/vancomycin; TLV: telavancin; DAP: daptomycin; QDA: quinupristin-dalfopristin; LNZ: linezolid. * Enrofloxacin (ENR) is not present in the list since it is restricted to veterinary use only. Note: the colours are those used by WHO and EMA to identify the categories of antibacterials.

**Table 3:** Resistance profiles with World Health Organization (WHO, AWaRe) and European Medicine Agency (AMEG) classifications. For each antibacterial and MDR, the prevalence of resistant profiles is reported.

WHO		HIA	HIA	HCIA		HPCIA	HIA	HCIA	HIA	HPCIA	HIA	HIA	HCIA	HCIA	HIA	HPCIA	HPCIA	HPCIA	HCIA	HIA	HCIA	
AwaRe		Access	Access	Reserve		Watch	Access	Watch	Access	Watch	Access	Access	Reserve	Access	Watch	Reserve	Watch	Reserve	Reserve	Reserve	Reserve	
AMEG		D	D	A	B	B	D	A	C	C	C	D	A	C	D	A	A	A	A	A	A	
Profile	Freq	P	OX	FOS	ENR	CIP	SXT	RD	C	E	DA	TE	TGC	CN	FD	CPT	TEC/VA	TLV	DAP	QDA	LNZ	MDR
A	1	**R**	**R**	S	**R**	**R**	**R**	**R**	**R**	**R**	**R**	**R**	S	**R**	S	S	S/S	S	S	S	S	**1**
B	7	**R**	S	S	S	S	S	S	S	S	S	S	S	S	S	S	S/S	S	S	S	S	0
C	3	**R**	**R**	S	**R**	**R**	**R**	S	S	**R**	**R**	S	S	**R**	S	S	S/S	S	S	S	S	**1**
D	2	**R**	**R**	S	**R**	**R**	**R**	S	S	**R**	**R**	**R**	S	**R**	S	**R**	S/S	S	S	S	S	**1**
E	6	**R**	**R**	S	**R**	**R**	**R**	S	S	**R**	**R**	**R**	S	**R**	S	S	S/S	S	S	S	S	**1**
F	3	**R**	**R**	S	**R**	**R**	**R**	S	**R**	**R**	**R**	**R**	S	**R**	S	S	S/S	S	S	S	S	**1**
G	2	**R**	**R**	S	**R**	**R**	S	S	**R**	**R**	**R**	**R**	S	**R**	S	S	S/S	S	S	S	S	**1**
H	2	**R**	**R**	S	**R**	**R**	**R**	S	**R**	**R**	**R**	**R**	S	**R**	S	I	S/S	S	S	S	S	**1**
I	2	**R**	**R**	S	S	S	S	S	S	S	S	S	S	S	S	S	S/S	S	S	S	S	0
J	1	**R**	S	S	S	S	**R**	S	S	S	S	**R**	S	S	S	S	S/S	S	S	S	S	**1**
K	1	**R**	S	S	S	S	S	S	S	S	S	I	S	**R**	S	S	S/S	**R**	S	S	S	**1**
L	1	**R**	**R**	S	**R**	**R**	**R**	S	S	**R**	**R**	S	S	**R**	S	**R**	S/S	S	S	S	S	**1**
M	1	**R**	**R**	S	**R**	**R**	**R**	**R**	S	**R**	**R**	**R**	S	**R**	S	**R**	S/S	S	S	S	S	**1**
N	1	**R**	S	S	**R**	**R**	S	S	S	**R**	**R**	**R**	S	S	S	S	S/S	S	S	S	S	**1**
O	1	**R**	**R**	S	**R**	**R**	**R**	**R**	S	**R**	**R**	**R**	S	**R**	S	S	S/S	S	S	S	S	**1**
P	1	S	S	S	S	S	S	S	S	S	S	**R**	S	S	S	S	S/S	S	S	S	S	0
Q	2	**R**	S	S	**R**	**R**	**R**	**R**	S	**R**	**R**	**R**	S	**R**	S	S	S/S	S	S	S	S	**1**
R	2	**R**	S	S	S	S	S	S	**R**	**R**	**R**	S	S	S	S	S	S/S	S	S	S	S	**1**
S	1	**R**	S	S	S	S	**R**	S	**R**	**R**	**R**	S	S	**R**	S	S	S/S	S	S	S	S	**1**
TOT.*		18	11	0	12	12	12	4	6	14	14	12	0	13	0	3	0	1	0	0	0	16
(%) *	-	94.7	57.9	0.0	63.2	63.2	63.2	21.1	31.6	73.7	73.7	63.2	0.0	68.4	0.0	15.8	0.0	5.3	0.0	0.0	0.0	84.2

Category abbreviations: HPCIA: Highest Priority, Critically Important Antimicrobials; HCIA: High priority, Critically Important Antimicrobials; HIA: Highly Important Antimicrobials. Antibacterial abbreviations: P: penicillin G; OX: oxacillin; FOS: fosfomycin; ENR: enrofloxacin; CIP: ciprofloxacin; SXT: trimethoprim-sulfamethoxazole; RD: rifampicin; C: chloramphenicol; E: erythromycin; DA: clindamycin; TE: tetracycline; TGC: tigecycline; CN: gentamycin; FD: fusidic acid; CPT: ceftaroline; TEC/VA: teicoplanin/vancomycin; TLV: telavancin; DAP: daptomycin; QDA: quinupristin-dalfopristin; LNZ: linezolid. * Total and percentage are referred to resistance profiles and not to strains. Note: the colours are those used by WHO and EMA to identify the categories of antibacterials.

**Table 4:** Reason for admission at the Veterinary Teaching Hospital of the dogs and cats from whom *S. pseudintermedius* strains were isolated. Data are reported in decreasing order of frequency.

Reason for Admission	Strain Freq. (Col. %)	Dogs	Cats
Orthopedic surgery	20 (50.0%)	18	0
Pyoderma or wound	8 (20.0%)	4	3
Ear infection	6 (15.0%)	5	0
Urinary tract infection	5 (12.5%)	3	2
Reproductive tract infection	1 (2.5%)	1	0
Total	40	31	5

**Table 5:** List of the 20 tested antibacterials and their classes/groups. The table reports the disk content (for ADD), the clinical breakpoint standards used for interpretation, and WHO, AWaRe and EMA AMEG classifications for each antibacterial.

Antibacterial Agent	Abbrev.	Class/Group	Disk Content (µg)	Clinical Breakpoint	WHO Classif.	AWaRe Classif.	EMA AMEG Classif.
penicillin G	P	β-lactamase sensitive penicillins	1 UI	EUCAST	HIA	Access	D
oxacillin	OX	β-lactamase resistant penicillins	1	EUCAST	HIA	Access	D
fosfomycin	FOS	phosphonic acids	50	CASFM	HCIA	Reserve	A
enrofloxacin	ENR	fluoroquinolones	5	CLSI Vet	-	-	B
ciprofloxacin	CIP	fluoroquinolones	5	EUCAST	HPCIA	Watch	B
trimethoprim-sulfamethoxazole	SXT	folate-pathway inhibitors	1.25/23.75	EUCAST	HIA	Access	D
rifampicin	RD	ansamycins	5	EUCAST	HCIA	Watch	A
chloramphenicol	C	amphenicols	30	EUCAST	HIA	Access	C
erythromycin	E	macrolides	15	EUCAST	HPCIA	Watch	C
clindamycin	DA	lincosamides	2	EUCAST	HIA	Access	C
tetracycline	TE	tetracyclines	30	EUCAST	HIA	Access	D
tigecycline	TGC	glycylcyclines	15	EUCAST	HCIA	Reserve	A
gentamycin	CN	aminoglycosides	10	EUCAST	HCIA	Access	C
fusidic acid	FD	steroid antibacterials	10	EUCAST	HIA	Watch	D
ceftaroline	CPT	anti-MRS cephalosporins	-*	EUCAST	HPCIA	Reserve	A
teicoplanin/vancomycin	TEC/VA	glycopeptides	-*	EUCAST	HPCIA	Watch	A
telavancin	TLV	glycopeptides	-*	EUCAST	HPCIA	Reserve	A
daptomycin	DAP	lipopeptides	-*	EUCAST	HCIA	Reserve	A
quinupristin-dalfopristin	QDA	streptogramins	-*	EUCAST	HIA	Reserve	A
linezolid	LNZ	oxazolidinones	-*	EUCAST	HCIA	Reserve	A

Category abbreviations: HPCIA: Highest Priority, Critically Important Antimicrobials; HCIA: High priority, Critically Important Antimicrobials; HIA: Highly Important Antimicrobials. Antibacterial abbreviations: P: penicillin G; OX: oxacillin; FOS: fosfomycin; ENR: enrofloxacin; CIP: ciprofloxacin; SXT: trimethoprim-sulfamethoxazole; RD: rifampicin; C: chloramphenicol; E: erythromycin; DA: clindamycin; TE: tetracycline; TGC: tigecycline; CN: gentamycin; FD: fusidic acid; CPT: ceftaroline; TEC/VA: teicoplanin/vancomycin; TLV: telavancin; DAP: daptomycin; QDA: quinupristin-dalfopristin; LNZ: linezolid. * MIC-test strips. Note: the colours are those used by WHO and EMA to identify the categories of antibacterials.

## Data Availability

Not applicable.
